# Utility, Limitations, and Future of Non-Human Primates for Dengue Research and Vaccine Development

**DOI:** 10.3389/fimmu.2014.00452

**Published:** 2014-09-24

**Authors:** Carlos A. Sariol, Laura J. White

**Affiliations:** ^1^Department of Microbiology and Medical Zoology, Caribbean Primate Research Center, University of Puerto Rico-Medical Sciences Campus, San Juan, PR, USA; ^2^Department of Internal Medicine, Caribbean Primate Research Center, University of Puerto Rico-Medical Sciences Campus, San Juan, PR, USA; ^3^Global Vaccine Incorporation, Research Triangle Park, NC, USA

**Keywords:** dengue, macaques, non-human primates, vaccine, genetic, neutralizing antibodies, cell-mediated immunity, animal model

## Abstract

Dengue is considered the most important emerging, human arboviruses, with worldwide distribution in the tropics. Unfortunately, there are no licensed dengue vaccines available or specific anti-viral drugs. The development of a dengue vaccine faces unique challenges. The four serotypes co-circulate in endemic areas, and pre-existing immunity to one serotype does not protect against infection with other serotypes, and actually may enhance severity of disease. One foremost constraint to test the efficacy of a dengue vaccine is the lack of an animal model that adequately recapitulates the clinical manifestations of a dengue infection in humans. In spite of this limitation, non-human primates (NHP) are considered the best available animal model to evaluate dengue vaccine candidates due to their genetic relatedness to humans and their ability to develop a viremia upon infection and a robust immune response similar to that in humans. Therefore, most dengue vaccines candidates are tested in primates before going into clinical trials. In this article, we present a comprehensive review of published studies on dengue vaccine evaluations using the NHP model, and discuss critical parameters affecting the usefulness of the model. In the light of recent clinical data, we assess the ability of the NHP model to predict immunological parameters of vaccine performances in humans and discuss parameters that should be further examined as potential correlates of protection. Finally, we propose some guidelines toward a more standardized use of the model to maximize its usefulness and to better compare the performance of vaccine candidates from different research groups.

## Current State of the NHP Model to Evaluate Dengue Vaccine Candidates, Usefulness and Limitations

Non-human primates (NHP) have been used to model a number of human infections and diseases, based on their genetic, physiological, and immune similarities with humans. In the case of dengue, NHP are the only natural vertebrate hosts besides humans susceptible to infection with the four serotypes of dengue viruses (DENV1–4). A number of species of NHP are infected in the wild by sylvatic DENV strains and experimentally by human clinical isolates without the need for virus adaptation [reviewed in Ref. (1, 2)]. A major limitation of this model for the study of dengue pathogenesis and vaccine efficacy evaluations is that the outcome of infection is subclinical in most instances (3), and does not recapitulate the symptoms seen in patients with dengue fever (DF) and dengue hemorrhagic fever and shock syndrome (DHF/DSS). Nevertheless, important similarities make it the best available model to evaluate vaccine immunogenicity and protective efficacy. Virus replication in NHP results in a peripheral viremia, with similar onset and duration to that reported in humans, although lower in magnitude. The induction of a robust neutralizing antibody (NAb) response and cellular immune responses also parallels the human immune response to dengue infection.

Two comprehensive reviews have been published recently on NHP as a potential model for dengue pathogenesis (2) and NHP infected with dengue in natural settings (1). Therefore, those topics will not be included here.

To date, in the absence of defined correlates of protection, the induction of NAbs and a significant reduction of post-challenge viremia in monkeys have been considered the closest predictors of vaccine immunogenicity and protective efficacy in humans. Therefore, the NHP model has been used for screening vaccine candidates, optimizing immunization strategies, and selecting the candidates with the best potential to work in clinical trials (4–7). Here, we present a comprehensive review of published studies on dengue vaccine evaluations using the NHP model (Table S1 in Supplementary Material). These include live-attenuated virus vaccines (LAV) attenuated by chimerization with yellow fever virus (8–15), by 3′ non-coding region (NCR) mutations (16–22), by chimerization with attenuated DENV (23–26), by serial passages in cell culture (27–29), by host range mutations (30, 31), or by mutations of a viral enzyme (32). Other vaccine platforms tested in NHP are DNA vaccines (33–39), inactivated virus vaccines (40–42), viral vectored vaccines (43–46), subunit protein vaccines (47–55), and prime/boost platforms (42, 43, 56, 57). A number of vaccine candidates tested in NHP have now been evaluated in humans, and data are available for safety and immunogenicity in phase I/II clinical trials (4, 19, 58–79), and efficacy for the leading vaccine candidate, Sanofi’s CYD1–4 (80, 81) (Table 1). In the light of clinical data, here we reassess and discuss the current state of the NHP model, its usefulness, limitations, and its predictive value, suggesting guidelines for the improvement of the model and the development of new reagents and standardized protocols.

**Table 1:** **Dengue vaccine candidates in clinical development: NHP and clinical studies**.

Vaccine type	Vaccine developer(s)	Pre-clinical studies in NHP		Clinical studies		
		Study type	Reference	Study type	Reference	Current Status
Live attenuated	WRAIR/GSK	MV (S, I)	(27)	MV (S, I) phase I	(59, 60, 69, 82)	Not being tested
		TV (S, I, P)	(29)	TV (S, I) phase I	(66, 79)	
		TV (I, P)	(28)	MV, TV (S, I) phase I	(78)	
				TV (S, I) phase I/II	(77)	
	Acambis/Sanofi Pasteur	MV (S, I, P)	(13)	TV (S, I) phase I	(68, 74, 76)	In phase III
		MV, TV (S, I)	(10, 11)	TV (S, I) phase II	(61, 70, 71)	
		TV (S, I, P)	(12)	TV (S, I, E) phase lIb	(81)	
		MV, TV (I)	(14)	(S, I, E) phase III	(80)	
		TV (I)	(8)	
	NIAID, NIH/Merck	MV (S, I)	(21, 22)	MV (S, I) phase I	(4, 19, 63–65)	In phase II
		MV (S)	(19)	TV (S, I) phase I	(62)	
		TV (S, I, P)	(16)	
		MV (S, I, P)	(17, 18)	
	CDC/Inviragen/Takeda	TV (S, I, P)	(26)	TV (S, I) phase I	(75)	In phase II
		TV (S, I, P)	(23)	
Inactivated virus	WRAIR/GSK	MV (I, P)	(41, 42)			In phase I
Subunit (rE)	Hawaii Biotech/Merck	MV, TV (I, P)	(51)			In phase I
DNA	NMRC	MV (I, P)	(33, 34, 36, 38, 39)	MV (S, I) phase I	(58)	In phase I
		TV (I, P)	(35)	

*MV, monovalent; TV, tetravalent; S, safety; I, immunogenicity; P, protection; E, efficacy in humans; WRAIR, Walter Reed Army Institute of Research; GSK, GlaxoSmithKline; NIAID, National Institutes of Allergy and Infectious Diseases; NIH, National Institutes of Health; CDC, Center for Disease Control; NMRC, Naval Medical Research Center*.

### Evaluation of attenuation and down-selection of monovalent components

One of the challenges of developing LAV vaccines is that each serotype component must be sufficiently attenuated relative to the parental strain, while maintaining adequate immunogenicity (NAbs). NHP have been used successfully to screen live-attenuated monovalent (MV) vaccine components for a balance between attenuation and immunogenicity. For example, Eckels et al. evaluated vaccine strains at different stages of attenuation by serial passages in PDK cells by screening for reduced viremia compared to parental strains while conserving immunogenicity (27). Down-selection from a number of starting candidates was achieved, and infectivity in NHP was useful to predict human reactogenicity and infectivity (82). Similarly, developers of a recombinant live attenuated dengue vaccine at the National Institutes of Allergy and Infectious Diseases (NIAID) used the NHP model to screen many MV live vaccine candidates attenuated by engineering specific attenuating deletions in the 3′NCR and/or by chimerization, ruling out several candidates that were either under- or over-attenuated, and identifying those with the most favorable attenuation and immunogenicity profile for further evaluation in clinical trials (4, 17, 18). Two recombinant DENV2 live-attenuated vaccine candidates (NIAID) with different degrees of attenuation have been characterized in both rhesus monkeys and humans for attenuation and NAb titers. The replication and immunogenicity patterns seen in NHP mimic closely those in humans (63), with one of the strains showing higher percentage of subjects with viremia and higher mean viremia titers (under-attenuated strain) in both species.

Although this model establishes general safety of dengue vaccine candidates, and can predict attenuation based on relative replication and viremia, the absence of human like symptoms limits its ability to show dengue-specific safety.

A number of non-replicating (based on DNA, inactivated virus, or subunit protein) and non-propagating (based on virus vectors) dengue vaccines candidates also have been tested in NHP for selection of antigens and antigen doses with best immunogenicity, and for the comparison and selection of vaccine adjuvants (Table S1 in Supplementary Material).

### Relative immunogenicity and serotype interference in tetravalent formulations

Another challenge for the development of dengue vaccines is the need to induce equivalent and long-lasting immunity to all four serotypes, due to the theoretical enhanced risk of severe disease if incomplete immunity is induced. The NHP model has been used to study serotype interference and relative immunogenicity of tetravalent (TV) vaccine components, guiding the identification of more balanced formulations and immunization strategies to minimize interference. In several instances, relative serotype dominance seen in NHP has been predictive of how the vaccine performs in the clinics, suggesting the potential for this model to predict when a candidate vaccine will induce unbalanced immunogenicity before going to clinical trials. Some examples are presented below. Guy et al. examined interference among the four serotypes of the CYD vaccine when present in equal concentrations (TV-5555) within the TV formulation in cynomolgus macaques (14) (Table S1 in Supplementary Material). NAb induced by each MV component was compared to that in the TV formulation. Interference was identified after the first and second immunizations, being serotype 4 the dominant and serotype 2 the weakest. Although each MV vaccine was immunogenic after one dose, the hierarchy of NAb titers observed for the MV vaccines was amplified in the TV mix (DENV4 > DENV1 > DENV3 > DENV2). Two phase I clinical studies in naïve human volunteers vaccinated with TV-CYD show dominance of serotype 4 as shown in NHP, while DENV1, a close second in NHP, induced the lowest titers in humans (74, 76). Monkeys receiving a second dose of CYD1–4 several months after the first dose developed a >fourfold anamnestic response to DENV1, 2, and 3, while NAbs to DENV4 were not boosted (14). Similarly, humans that received a second dose of the same vaccine CYD1–4 six months after the first one, produced anamnestic NAbs to DENV1, 2, and 3 but not to DENV4 (74), suggesting in both cases a robust sterilizing immunity to the dominant serotype 4 component. Potential approaches to minimize serotype interference were identified in macaques, by either separating the delivery of the four components in time and in anatomical sites of injection, or by adjusting the doses of each component (14). However, the lack of protection data from this study has limited its utility.

Serotype interference was also identified in different formulations of the LAV DENVax developed by CDC/Inviragen/Takeda when tested in cynomolgus macaques. When the components were present at the same concentrations, either low (TV-3333) or high (TV-5555), serotype 2 was the strongest and serotype 4 was the least immunogenic after two immunizations. By adjusting the relative concentrations (TV-3355), the immunogenicity of DENV4 was improved (26) (Table S1 in Supplementary Material). Results from ongoing phase I clinical trials (high and low doses) indicate that NAb levels against DENV2 are the highest and NAb to DENV4 are the lowest of the four serotypes in humans, as seen in macaques (23, 75). In a third example, the TV-3 formulation of the LAV vaccine developed by NIAID (LATV), resulted in robust NAbs to all 4 serotypes after a single immunization both in rhesus macaques (16) and when tested in flavivirus-naïve adult volunteers (62).

While these examples show some similarities between vaccine performances in NHP and humans, there are instances when the data do not agree, and it is unclear at this time whether these differences are the result of variability due to the small sample sizes, or to more fundamental differences between NHP and humans. As more data becomes available from clinical studies, these issues will hopefully become clearer.

### Assessing immunogenicity in NHP

#### NAb responses to infection and vaccination in NHP

Although NAbs to flaviviruses, including dengue, have been considered necessary to prevent infection and/or disease, and required for vaccine efficacy (6), researchers are re-examining whether Ab is the best predictor of protection, and how to best measure antibody-mediated neutralization *in vitro*. This has been prompted by recent clinical findings where protection against DENV2 infection was not provided despite the presence of high NAb levels (80, 81).

Similarities in the humoral response after primary and secondary dengue infections between NHP and humans support the use of this model for evaluations of vaccine-induced Ab responses. Like humans with primary dengue infection or immunized with a MV vaccine, a number of NHP species can mount robust long-lasting serotype-specific NAbs responses that prevent re-infection with the same serotype, and short-lived cross-protective NAbs (83–88). Koraka et al. showed that the level and duration of viremia, and the kinetics and magnitude of Ab responses observed in experimentally infected macaques were similar to those observed in most uncomplicated human dengue infections, and that Abs measured were largely cross-reactive (88). Upon secondary infection, or immunization with multivalent vaccines, NHP show a broad NAb response to multiple serotypes (88). Secondary infection with DENV3 following a DENV1 infection suggested the phenomenon of original antigenic sin (89) in macaques for that sequence of infections (88).

Recent studies point to potential similarities in the quality of the induced NAbs in NHP and humans. In-depth characterization of the primary response in NHP reveals that unlike mice but similar to humans (90), NHP produce serotype-specific NAbs that predominantly bind to sites other than domain III on the E glycoprotein (EDIII) (46). Interestingly, there is evidence that the nature of the DENV antigen modulates the NAb targeting. For example, soluble E ectodomain expressed using an alphavirus-vectored vaccine (VRP) induces in NHP NAbs that bind predominantly to EDIII, while VRP expressing prME subviral particles induce in NHP predominantly non-EDIII binding antibodies (46). A recent study on epitope targeting using an epitope transplantation approach showed that the E domain I/II hinge region of DENV3 and DENV4 is the primary target of long-term, serotype-specific NAbs in humans and in rhesus macaques after primary infection (91). One implication of these observations is that the quality of the NAb response in NHP and humans may be different between vaccines based on live-attenuated virus or inactivated virions and protein subunit vaccines, and the NHP model may be able to predict these specificities. These different vaccine platforms need to be tested in NHP and in humans in order to help answer the question of what region(s) on E protein are targeted by NAbs induced by a successful dengue vaccine.

The genotypic breadth of vaccine-induced NAb responses is an important Ab quality to examine, since some vaccine antigens may have a narrower set of serotype-specific epitopes that may affect the breadth of the protective immunity. Recent studies have addressed the breadth of vaccine-induced NAbs in NHP (8, 16, 46).

#### NAbs and protection from viremia in NHP

**Figure 1:**
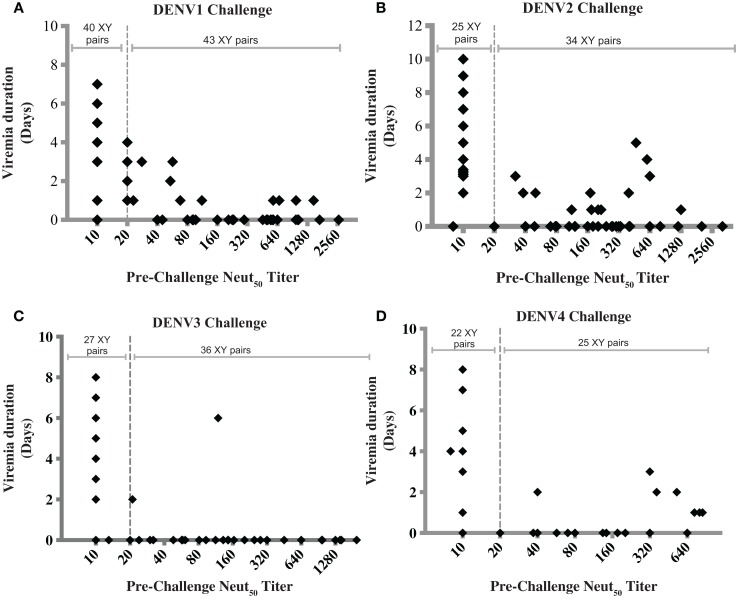
**Correlation between pre-challenge Neut_50_ titers and duration of post-challenge viremia**. Each data point was obtained from 1 of 10 published studies on dengue vaccine candidates tested in NHP. Only studies that reported Neut_50_ titers and duration of viremia for individual monkeys were included, and for each study, data from both immunized and unimmunized controls were used [Men et al. (44), Guirakhoo et al. (12), Sun et al. (29), Chen et al. (43), Raviprakash et al. (45), Bernardo et al. (49), Clements et al. (51), Osorio et al. (26), Maves et al. (40), White et al. (46)]. Data were combined based on the challenge virus serotype into graphs **(A–D)**, corresponding to serotypes 1–4, respectively, regardless of vaccine type or whether it was monovalent or tetravalent. Dotted line indicates a Neut_50_ titer of 20. Titers below the limit of detection were given a value of 10, whether the limit of detection was 10 or 20. The number of XY pairs with Neut_50_ titers <20 or ≥20 are indicated at the top of each graph. Pearson correlation coefficients (*r*) were −0.4453, −0.3367, −0.4182, and −0.3063 for graphs **(A–D)**, respectively.

The ability of NAbs to mediate protection in NHP has been shown by passive transfer experiments (92, 93). Hahn et al. showed that the infusion of a bivalent monoclonal antibody in NHP was followed by subsequent clearance of dengue virus from the vascular system (92). Lai et al. demonstrated protection against DENV4 challenge in rhesus monkeys by passively transferred humanized monoclonal antibody (93). In most vaccine studies in NHP, the presence of pre-challenge NAbs seems to correlate with reduction or absence of post-challenge viremia. However, a threshold titer that correlates with protection in NHP has not been identified, and there are reports of animals with moderate to high Neut_50_ titers that show breakthrough viremia (12, 26, 29, 45, 46). To determine whether combining and analyzing data from several studies would provide additional insight, we collected data from 10 published studies of dengue vaccine candidates in NHP, and used them to graph pre-challenge Neut_50_ titers vs. duration of viremia (Figure 1). Four graphs were generated, Figures 1A–D, corresponding to protection from viremia after challenges with dengue serotypes 1–4, respectively. Only studies that reported Neut_50_ titers and duration of viremia for individual monkeys were included, and for each study, data from both immunized and unimmunized controls were used (12, 26, 29, 40, 43–46, 49, 51). A strong negative correlation between pre-challenge Neut_50_ titers and duration of viremia was observed, with Pearson correlation coefficients (r) of −0.4453, −0.3367, −0.4182, and −0.3063 for serotypes 1–4 challenges, respectively. A high percentage of animals with titers = or >20 had no viremic days after challenge (77% for DENV1, 76% for DENV2, 94% for DENV3, and 72% for DENV4 challenges).

Figure 1 also shows breakthrough viremia for some animals with Neut_50_ titers >20, although in most cases the viremia was of shorter duration. The number of monkey with viremia out of those with titers of 20 or higher were 10 out of 43 (23%) after DENV1 challenge, 10 out of 41 (24.4%) after DENV2 challenge, 2 out of 36 (5.5%) after DENV3 challenge, and 7 out of 25 (28%) after DENV4 challenge. An interesting finding is that the proportion of breakthrough viremia cases after DENV3 challenge was lower (5.5%) compared to the other three serotypes, suggesting that a surrogate of protection may be serotype-specific.

The interpretation of these combined data is limited by the use of a single readout of protection, duration of viremia, and because different methods to measure viremia were employed. In addition, differences in the challenge strains and doses probably added variability to the data, which is evident in the broad range of duration of viremia in unvaccinated controls (1–8 days). Also, the data includes MV and TV vaccine studies. In the case of TV vaccine studies, there are differences in the time between the last immunization and the challenge, which may have affected the levels of short-lived heterotypic, potentially protective NAbs at the time of challenge. However, only homologous Neut_50_ titers are used in the analysis.

In spite of these caveats, examining the collective data in this way suggests a strong correlation between NAb titers and protection in the NHP model, but also indicate that NAb titers >20, as measured *in vitro* on epithelial cells, not always prevent challenge virus replication in the NHP model. This analysis raises interesting questions. For example, could some cases of breakthrough viremia be explained by the induction of a qualitatively distinct group of NAbs that can neutralize infection of epithelial cells *in vitro* but not infection of target cells *in vivo*?

On the other hand, protection from viremia has been shown in rhesus macaques with poor NAbs (41, 94) or after NAbs have waned (39), suggesting that other immune mechanisms may play a role in protection, especially when Abs are suboptimal. This is supported by studies in mice, where cellular responses were sufficient to protect from lethal challenge (95).

#### Current *in vitro* neutralization assays

Most DENV vaccine pre-clinical and clinical studies reported to date examine neutralizing activity in serum by measuring *in vitro* neutralization on epithelial cells of animal origin (LLC-MK, BHK-21, Vero), using the standard dengue PRNT assay, originally described by Russell (96), and recommended by the World Health Organization (WHO) (97) (Table S1 in Supplementary Material). Although PRNT is considered the gold standard, its use is not standardized among labs, and protocol variations in cell lines, PRNT end point titers, virus passage number, and presence of complement are known to affect the Ab titer readout (98, 99). Variations of the PRNT have been developed for higher throughput, reduced duration and labor, to test against DENV clinical isolates that do not plaque well, and to measure neutralization in more biologically relevant cells, like primary human myeloid cells. Alternative assays include ELISA-based microneutralization (ELISA-MN) (100, 101), flow cytometry-based assay using Vero cells, or DC-like cells (46, 102, 103), assays based on FcγR-bearing human cells (104–106), and a reporter virus based system (107). A comparative evaluation of MN and DC assays vs. PRNT indicated that the assays are not always in agreement (106). Recent studies show that NAbs measured on epithelial cells result in different titers compared to assays that use FcγR-bearing cells (105, 108, 109).

There is an urgent need for determining what *in vitro* neutralization assay(s) best correlate with protection in NHP and in humans, and to minimize assay variation and experimental inconsistencies in the *in vitro* neutralization assay, which have made it difficult to compare NAb titers among studies.

It is important to note that currently used neutralization assay cannot determine whether a TV response is generated by four serotype-specific responses or from cross-reactive short lived and less protective Abs. Therefore, only by allowing cross-reactive short-lived antibodies to decay, and confirming TV responses after 6–12 months, will the assay measure truly serotype-specific NAbs.

#### Cell-mediated immunity

To date, cell-mediated immunity (CMI) is not required for clinical evaluation of dengue vaccine candidates. However, after results of the first efficacy study in humans, there is increased interest in measuring CMI in vaccines tested in clinical trials due to their potential role in protection (81, 110). There have been a few studies addressing CMI in NHP after DENV infection and vaccination (Table S1 in Supplementary Material). These studies have examined the induction of dengue-specific cytokine-producing cells from PBMC stimulated with purified DENV or NS1, NS3, or NS5 peptide pools, using ELISPOT or intracellular cytokine staining (ICS) or cytotoxicity assays (23, 25, 26, 28, 35, 39, 43, 45, 50–52).

Cell-mediated immunity after primary, secondary, and tertiary experimental infection in cynomolgus macaques has been examined by Koraka et al. (88). Bulk T-cell-mediated responses were found against homologous and heterologous viruses even after primary infection. T-cell-mediated IFNγ production measured after secondary DENV3 infection following a DENV1 infection suggest a phenomenon of original antigenic sin, as described after human infections (111). Mladinich et al. (112) studied the kinetics of DENV specific T cells in rhesus macaques after primary infection with DENV2, showing multifunctional CD4^+^ and CD8^+^ T cells specific for NS1, NS3, and NS5. These studies suggest that infection of NHP with DENV result in CMI that are similar to those in humans in kinetics and serotype-specificities (110, 113), and suggest that NHPs may be a useful model to further understand the cellular responses to vaccine candidates and their role in pathogenesis (88, 112).

Vaccine-induced cellular responses have been studied in NHP for a few vaccine candidates. Osorio et al. (26) reported that macaques immunized with TV live-attenuated vaccine DENVax showed robust numbers of DENV-2-induced IFNγ and IL-2 secreting cells by ELISPOT, when peripheral blood mononuclear cells (PBMCs) were stimulated using semi-purified concentrated wt DENV-2. Consistent cytokine-secreting cells stimulated by DENV1, DENV3, or DENV4 were not observed (26). A study by Ambuel et al., using ICS and stimulation with NS1, NS3, and NS5 peptide pools, reported that the same DENVax vaccine-induced CD4^+^ and CD8^+^ T cells producing IFN-γ, IL-2, and TNF-α and targeted the DENV2 NS1, NS3, and NS5 (23). Human T cell data from clinical trials is pending. A DNA vaccine developed by the Naval Medical Research Center (NMRC), D1ME100 has been tested in NHP and in phase I trials (Table S1 in Supplementary Material). Chen et al. reported dengue-specific T cell responses in cynomolgus macaques immunized with three doses of a plasmid DNA vaccine expressing DENV1 prME (43). The cellular responses were measured by stimulating PBMC from immunized animals with purified DENV1 and measuring IFNγ secreting cells by ELISPOT. When the prototype DENV1 DNA vaccine D1ME100 was tested in humans at high and low dose using biojector2000 to deliver, T-cell IFNγ responses were detected after three immunizations in 50 and 83% of subjects in the low (1 mg) and high (5 mg) dose groups, respectively (58).

In summary, measuring CMI is complex and requires careful sample collection and storage, and standardization of assays and antigens used to stimulate immune cells. Like the NAb assays, CMI assays lack standardization of critical reagents and methods. Semi-purified DENV as stimulating antigen does not result in optimal T-cell responses. While peptide arrays for a subset of viral proteins and serotypes are now available through BEI Resources, other antigens/peptide arrays should be developed and validated. Tracking specific CD4^+^ and CD8^+^ T-cell responses has been difficult due to a lack of mapped epitopes and associated reagents within the macaque model. Mapping DENV specific CD4^+^ and CD8^+^ T-cell responses in NHP and producing MHC:peptide tetramers would greatly advance this model and accelerate the pace of dengue virus vaccine development. The availability of these reagents would allow performing more in-depth analysis of vaccine-induced CD8^+^ T-cell responses that may be critical for the success of future dengue virus vaccines.

### The NHP dengue challenge model

In the absence of dengue-induced disease in NHP, viremia is measured post-challenge as a surrogate of protection. Even though it is not required by the Food and Drug Administration (FDA), to date all dengue vaccine candidates in clinical trials have previously shown efficacy in NHP (114). However, results of the lack of protection by the CYD1-4 vaccine candidate in the first phase IIb efficacy trial (81), and lack of protection against serotype 2 in the first phase III efficacy trial (80) raise questions of whether the NHP challenge model is able to predict efficacy in humans. Unfortunately, the DENV challenge model in NHP is not standardized, making it difficult to compare the results from studies performed by different studies (Table S1 in Supplementary Material). We discuss below how specific limitations of the NHP challenge model may affect its performance.

#### Diversity in NHP used for Dengue studies

A number of NHP species used to study dengue pathogenesis and vaccines evaluations are listed in Table 2, along with information on the relative frequency with which they have been reported, the number of MHC alleles known and sequenced to date for each species, and an estimated number of reagents and/or protocols available through a free resource tools in support of research.

**Table 2:** **Species of NHP used in dengue pathogenesis and vaccine studies**.

Common name	Scientific name	Percent of published reports (1,178 total) (%)	MHC designation/no. alleles available^a^	Number of available resources (antibodies, cell lines, SOPs) (NIH non-human primate reagent resource)^b^
Rhesus macaques	Macaca mulata	51.35	Mamu/1,197	221
Green monkeys	*Cercopithecus aethiops*	34.80	*Chsa*/64	38
Cynomolgus macaques	*Macaca fascicularis*	11.62	Mafa/1,506	202
Patas monkeys	*Erythrocebus patas*	1.0	N/A	N/A
Yellow baboons	*Papio cynocephalus*	0.6	*Papa*/30	N/A
Japanese macaques	*Macaca fuscata*	0.33	*Mafu*/27	N/A
Mangabeys	*Cercocebus spp*	0.1	N/A	193
Others		1	N/A	N/A

*^a^Major histocompatibility complex genes of non-human primates. Available from the immuno polymorphism database (IPD), European molecular Biology Laboratory, and European Bioinformatic Institute*.

^b^www.nhpreagents.org

The use of multiple species makes it difficult to integrate results from different studies. In addition, within the same species, the genetic composition and variability in the genetic background of animals in the study can have an impact on the results (115). It has been well documented that the geographic region from which the founder animals are derived determines the MHC haplotype composition of the population (116, 117). Such genetic differences may also contribute to the phenotypic variance of pre-clinical trials when animals from different locations are included as experimental subjects, or when animals from different countries are compared. High inter-animal additive genetic variances increase phenotypic variance in full-breed animals and can obscure correlates under study (116, 117). Theoretically, this may be particularly relevant when addressing the role of cellular-mediated immune responses, but no data are available yet. On the other hand, as shown in Table S1 in Supplementary Material, six species of NHP have been used to study vaccine-induced immunogenicity, and NAbs have been reported in all of them. In addition, the same vaccine candidate has been tested in two species of monkey for two vaccine platforms, CYD by Sanofi (10, 14), and DNA by NMRC (34, 39), and no major differences in NAb responses can be attributed to the monkey species used. However, in most studies in this report, large animal-to-animal variation among NAbs titers exists even among animals in the same experimental group.

The more common species in use in biomedical research, rhesus macaques, can be very diverse in its genetic background, with groups of animals with Indian origin clustering more close with animals belonging to western China than to the other Indian group, consistent with the hypothesis that they originated in Burma, which is very close of western China (117). It has also been proposed that a natural gene flow among populations of Chinese (and Burmese) rhesus macaques and Vietnamese cynomolgus macaques (*Macaca fascicularis*) exists (115, 117, 118).

Being of intermediate genetic composition, admixed animals may respond very differently to experimentation involving traits that have a phenotypic variance that is lower than either unmixed parent population due to increased heterozygosity and stabilizing selection. The Cayo Santiago colony at the Caribbean Primate Research Center (CPRC) is an example of a population of rhesus macaques used in biomedical research in the US in which all the animals are derived from founders from the Indian subcontinent and it has been demonstrated that this population is genetically homogenous and unadmixed (117). Populations of cynomolgus macaques, the second most frequent species used for dengue studies can be even more genetically divergent as they may have originated from different locations in Southeast Asia including Sumatra, Corregidor, Mauritius, Singapore, Cambodia, Zamboanga, and others. Genetic variations among regional populations of cynomolgus macaque are the main cause of differences in research subjects that determines the repeatability of experimental outcomes (115). While the impact of the genetic diversity among and within the used species on DENV replication has not been addressed carefully, there have been reports of animals that can be considered as natural viremia controllers (29, 67, 93, 119–121).

Genetic homogeneity among research animals allows for the utilization of fewer subjects and cost-effective research projects. The origin ancestry and pedigree of the dengue research subjects should be taken in to account. It has been proposed that to maximize resolution of experimental treatment effects, animals of unmixed ancestry with paired coefficients of relationship below that of first cousins (*r* < 0.125) should be used (115). These considerations have important implications on what should be the minimum number of monkeys per experimental group. Supported by published data and in our own experience, cohorts should include at least six and not less than four, to minimize the impact of the genetic variability in the results.

#### DENV challenge strain, dose, and route

A comprehensive review of DENV strains used to infect different species of NHP and the viremia they caused can be found in Clark (2) and Hanley (1). Most vaccine studies in NHP include a challenge with one or more DENV serotypes to assess protective efficacy. The challenge strains used vary among studies, including wild-type viruses, near wild-type viruses or homologous viruses parental to the vaccine strains (Table S1 in Supplementary Material). To date, there is not a repository stock of DENV strains for each serotype available to different research groups, nor a standardized challenge protocol. These are in urgent need in order to guarantee the reproducibility and comparability of the results. Even using same strains by different groups has shown different outcomes. Hickey et al. (86) were unable to confirm viremia in groups of four rhesus macaques challenged with strains of dengue 1 and dengue 4 shown to induce detectable viremia in rhesus macaques in different protocols conducted by other groups (16, 46). This confirms that in addition to the virus strain, dose, and route of administration, other variables like the challenge virus passage history and the time post-challenge for sample collection, among others, need to be considered.

To determine protective efficacy, the dose of the challenge virus has to ensure a measurable and reproducible viremia for several days. Only a limited number of studies have attempted determining the infectious dose delivered during natural dengue infection. One study suggests that the amount of infectious particles transmitted by *A. albopictus* ranges from 1 × 10^4^ to 1 × 10^5^ plaque forming units (PFU) (122).

To our knowledge, the only study comparing inoculum dose and viremia in NHP was performed by Halstead et al. (123). In that study, animals were challenged with low (8–50 PFU) and high doses (5 × 10^3^–5 × 10^5^ PFU) of DENV, which resulted in levels of viremia ranging from 0.6 to 1.8 × 10^3^ PFU/ml. In general, doses from 1 × 10^4^ to 1 × 10^5^ PFU, independent of the route, have shown to be enough to induce detectable viremia and seroconversion in most of the animals, or strong seroconversion in spite of absence of viremia [reviewed in Ref. (124)]. Higher doses do not seem to result in higher viremia. Even doses of 1 × 10^7^ PFU delivered via intravenous injection, resulted in peak viremia of 8 × 10^3^ PFU/ml (3). In addition, a significant negative relationship between challenge dose and duration of viremia has been documented (125).

#### When to challenge and protection readouts

The time allowed between the last immunization and the challenge varies from 30 days to 12 months in reports of dengue vaccine studies in NHP (Table S1 in Supplementary Material). These differences make it difficult to compare vaccine efficacy among vaccine candidates from different studies. There seems to be consensus in the field to allow at least 6 months between the last immunization and the challenge. This would increase the stringency of the challenge and better assess protection mediated by serotype-specific, long-term protective immunity, minimizing protection readouts mediated by short-term heterotypic immunity. In humans, heterotypic protection after primary infection lasts for a few months (126). NHP, like humans, develop long-term serotype-specific NAbs that last as long as the duration of the study, 13 (86) or 24 months (91), and shorter-lived cross-reactive NAbs. Hickey et al. reported that the duration of the heterotypic NAbs varied among serotypes and among animals; these NAbs were mostly absent after 120 days in a DENV3 infection, while decreased at different rates between 120 and 390 days in animals infected with the other serotypes (86). Messer et al. confirmed the presence of homotypic NAbs up to 24 months after infection with DENV3 or DENV4, and also showed detectable NAbs (Neut_50_ titer = 35) against the heterologous serotype in one out of four animals 24 months after infection (91).

The major limitation of the NHP dengue challenge model is that protection from low-level viremia in NHP may not reflect protection from DF or DHF in humans. In the absence of better defined correlates of protection, different readouts of protection in the NHP model have been proposed: (1) prevention of infection (sterilizing immunity), measured by absence of viremia, and absence of anamnestic response, as defined by fourfold increase in NAb titer. (2) Significant reduction in the duration and magnitude of the viremia (infectious virus or RNA genome equivalents) without preventing an anamnestic response, where a robust anamnestic response is indicative of a robust memory response with potential to prevent disease. To date, there is not clear evidence that the more stringent criteria for solid immunity in NHP is what correlates with protection in humans. Most vaccines tested in NHP show some level of anamnestic responses (Table S1 in Supplementary Material), suggesting non-sterilizing immunity. Sterilizing immunity has been reported after DENV1 MV NIH LAV vaccination and challenge (22), and after one dose of LATV followed by DENV4 challenge (16). Viremia has been measured by different methods. A semi-quantitative measurement involves amplification of the virus collected in the blood in insect or mammalian cells followed by IFA (34). Quantitative determinations of infectious virus particles are done by plaque assays and immune-focus assay (19, 26, 46, 86, 87, 91). A quantitative RT-PCR has been used to determine RNA genome equivalents (20, 32, 48, 86, 88, 121, 127, 128). However, components in the monkey sera can inhibit non-specifically the RT-PCR assay, rendering false negatives or amplifications that are difficult to interpret, even when using virus stocks that have been shown to induce productive infection in NHP measured by other methods (83, 86, 120). In addition, time to viremia was significantly shorter, and duration of viremia was significantly longer when measured by RT-PCR compared to plaque forming units (125). Based on these results, it is recommended that more than one method be used to characterize and quantify post-challenge viremia.

#### Toward a NHP disease model

Although most dengue infections in NHP are subclinical, cutaneous hemorrhages, and lymphadenopathy have been reported (2, 3, 123, 129–131), and only a small fraction of studies have reported rashes post infection (3, 129). Onlamoon et al. showed recently that by using an i.v. route and a high dose of 10^7^ PFU, a primary infection in rhesus macaques with DENV2 strain 16681 resulted in cutaneous hemorrhages, and suggested that further amplification of disease severity could result from refining other parameters like virus strain, factors from infected mosquito saliva, and macaque genetic factors (2, 3). Although such manipulations of route and dose of infection distant the model from naturally occurring dengue, further exploring this disease model may help understand the impact of dengue infection on a set of particular cells playing a key role in pathogenesis in a higher animal model.

Another potential factor that may modulate dengue infection and the course of disease is mosquito’s saliva. There are a number of studies supporting its modulatory role on the immune response to dengue virus (132–137) and other arboviruses (138–145). However, its potential role modulating disease in NHP has not been examined. Studies comparing the course of infection and immune response between viruses delivered into the skin by needle and by mosquito bite should be done. Until then, we could be missing the role of an important component defining the quality of the immune response to dengue in nature and overlooking potential key data from the pre-clinical dengue vaccine trials.

#### Sequential infections modeling ADE

Early work by Halstead et al. showed that after a secondary DENV infection in NHPs viremia increases, suggesting that ADE may increase viral load through cross-reactive Abs (129, 146). However, because few numbers of animals were included in each experimental group, only a trend was reported but no significant differences were established.

A cross-reactive response with the highest Ab titers directed against the primary infecting serotype (and potential ADE induction) was showed only when the sequential infection was DENV1–DENV3 but not when DENV4 was the primary infecting serotype (87, 88). *Aotus nancymae* monkeys sequentially infected with dengue 1 followed by dengue 2, either with an American or an Asian genotype, did not showed any significant viremia increase, ruling out ADE mechanism for this particular sequential infection (87). Recently, it has been showed that primary infection with serotypes 1, 2, and 4 but not serotype 3 induces long-lasting cross-reactive neutralizing antibodies in NHP. However, as these antibodies decrease after 120 days of infection, the host may become susceptible to develop ADE after DENV1, 2, or 4 but not DENV3 infection (86). The passive transfer of anti-DENV monoclonal Ab 1A5 prior to DENV infection resulted in a viremia increased 3- to 100-fold in RMs after (147). A previous study had shown that the administration of polyclonal diluted dengue 2 immune cord-blood serum to few animals resulted in increased viremia after infection with dengue 2 (148). The data reviewed here confirms that the role of ADE in NHP infection is still controversial. However, due to the impact of ADE in dengue pathogenesis (149) and its implications in the quality of the immune response elicited after vaccination, NHP should continue to be explored as a potential contributor to the understanding of the role of ADE in dengue pathogenesis.

## The Future of the NHP Model

The value of the NHP model for dengue research has the potential to be of larger scope than to date.

### In-depth characterization of immunity to vaccination

A better characterization of the immune response to vaccination may help define better correlates of protection. These include mapping where NAbs bind, measuring early type-specific antibody secreting cells (ASC), Ab avidity, and neutralization in FcγR-expressing cells. Studies should include genotypic breadth, contribution of type-specific vs. cross-reactive antibodies, epitope repertoire, ADCC, role of complement, and cellular response, including multifunctionality of CD8^+^ T cells. Recent studies have shown the plasticity of this model allowing the replication of chimeric virus carrying transplanted EDI–EDII hinges (91), and at the same time, confirming the value of the model for a better mapping of the antibodies repertory *in vivo* (46, 91).

### Modeling vaccination in dengue endemic regions

Most dengue vaccines tested in NHP use dengue naïve animals, and in a few cases animals immune to yellow fever. Since most vaccines will be used in dengue endemic countries, studying how vaccines perform in monkeys after passive transfer of dengue Abs or after previous wild-type dengue virus infection will be of great value.

### NHP model validation by clinical studies

The predictive value of the NHP model for candidate vaccine efficacy is limited by the lack of efficacy data available from studies of parallel vaccines in humans, lack of standardized protocols and reagents, and lack of in-depth immunological studies in primates and humans to infection and vaccination. As phase III clinical trial data becomes available, and as human challenge is incorporated into early clinical testing (150), comparison of results from ongoing clinical evaluations and NHP studies will lead to validation and improvement of the NHP model, to become a better predictor of the human response to vaccination and become a more robust model, reducing the need to do human challenges. For this to happen, results must be shared promptly among vaccine developers and the dengue research community, to expedite and facilitate the identification of better correlates of protection.

### Testing modified MAbs as therapeutics

From therapeutic point of view, vaccines have been the dominant if not the unique approach intended in NHP to fight dengue virus infection (4–6, 46). However, other therapeutic approaches like the use of MAbs are almost unexplored. Recently, it has been shown the effectiveness of using MAbs to control dengue infection in mice, and *in vitro* methods have been developed that predict the ability of modified MAbs to act therapeutically against antibody-enhanced disease *in vivo* (151). The potential of these therapeutics tools in humans and alternatives for improvement have been extensively reviewed (152). However, few studies have been conducted in NHP. Hahn et al. showed that the infusion of a bivalent monoclonal antibody in NHP was followed by subsequent clearance of dengue virus from the vascular system (92). Passive i.v. transfer of antibody IgG 5H2ΔD protected monkeys against DENV-4 infection and this was confirmed by absence of both viremia and specific anti-dengue antibodies (93). These studies showed the feasibility of using NHP to test and to develop those new therapeutic alternatives. Furthermore, over the past 3 years, we have learned that NAb responses in mice and people target different domains on DENV particles (EDIII vs. EDI–EDII hinge) (46, 90, 153–156). Moreover, our group has confirmed that NHP exposed to natural DENV infections appear to develop neutralizing Abs responses that are qualitatively similar to the human response (46, 154). For this reason while mice continue to be a very useful model for screening, the final functional significance of therapeutic MAbs should be tested in macaques (90, 154, 155). Although they could be expensive, therapeutic MAbs would help saving lives if they are used in the first hours/days after the infection in those patients where severe manifestations can be anticipated using the WHO guidelines for dengue case classification (157).

Another alternative therapeutic approach is the use of Toll-like receptors (TLR) agonist in vaccine formulations or anti-viral strategies against different pathogens. TLR have been already assayed for other viruses (158–164). However, so far only one study addressed the impact of TLR agonists on dengue virus immune response *in vivo* in NHPs (121). This work and the fundamental role of the TLRs in modifying the immune/vaccine response guarantee further studies on the impact of TLRs in dengue pathogenesis/vaccine studies.

### Standardizing the use of the NHP model

Based on recommendations compiled in the literature (114, 165, 166) and from discussions with colleagues in the field, the following guidelines toward standardization are proposed.

#### Animal selection

- Select healthy animals with a homogenous genetic background (coefficients of relationship below *r* < 0.125).
- Most published studies using four to six animals per experimental group have yielded interpretable data. However, in many cases, increased statistical rigor is needed. The minimum number of animals per group to have adequate statistical power should be calculated for each specific objective (167).

#### Evaluation of immunity

- Until an improved and more predictive *in vitro* neutralization assay is developed and validated, the WHO recommended that a standard plaque-reduction (PRNT) or focus-reduction neutralization assay should be used to evaluate vaccines in NHP, using optimized protocols and reagents and reference virus strains representing different serotypes and genotypes.
- Evaluation of CMI is strongly recommended as part of the analysis of vaccine immunogenicity in NHP. Standardized procedures for collecting, processing, and storing of PBMC should be used. PBMC should be collected before vaccination to establish a baseline, and then at various time points after vaccination to measure effector function, memory, and durability. Stimulating antigens should be well-characterized and available to the dengue community.
- Post-challenge NAbs should be measured, to determine whether the vaccine induces anamnestic responses and sterilizing immunity. This information will be of value once phase III clinical data is available.
- When possible, each monotypic component of the vaccine should be tested alone and combined in the TV formulation, to evaluate serotype interference.

#### Dengue challenge and protection

- A collection of well-characterized challenge viruses should be available to all researchers as a publicly funded repository, such as BEI resources. Researchers with strains that replicate well in NHP should contribute their strains to this collection.
- Post-challenge viremia should be determined by at least two different methods, one of them being a plaque- or foci-based assay for infectious virus. Universal techniques should be adopted.
- Challenge should be done at least 6 months after the last immunization, using homotypic wild-type virus strains that have been well characterized previously and result in sustained and consistent viremia for several days. The doses of challenge virus to obtained consistent viremia should be pre-determined, and generally ranges between 10^4^ and 10^5^ PFU.

## Ethics and Humane Use of NHP

For most candidate human vaccines, including those for dengue virus, immunogenicity, and if possible protective efficacy of the candidate formulation, has to be shown in a relevant animal model before it is tested in humans. There is no *in vitro* correlate of *in vivo* immunogenicity and protective efficacy for candidate dengue vaccine formulations. Therefore, animal models must be used in the development of dengue vaccines to screen potential candidates and allow those that show robust immunogenicity to move forward into clinical trials. Most of the work reviewed here included ethical statements on the humane use of animals.

Authors strongly encourage all researchers working with NHP to follow the local regulations on the use of NHP for research. Also, we support the implementation of additional steps to ameliorate suffering in accordance with the recommendations of the Weatherall report, “The Use of Non-human Primates in Research.” It is also advised to have animals under an environmental enrichment program approved by the local committee.

## Conclusion

To date, NHP data have been the gatekeeper for vaccines advancing to clinical trials, based on neutralizing activity in serum and reduction of post-challenge viremia, which indicates to vaccine developers and regulatory agencies of the potential for efficacy in humans. Recent clinical data has become available to compare vaccine performance in NHP and humans. Such analysis indicates that the replication and immunogenicity of vaccines tested in NHP has parallels to the human responses to the same vaccines, specifically regarding under-attenuation, relative serotype dominance, and immunogenicity in TV formulations, induction or not of anamnestic responses upon second vaccine doses and seroconversion to all four serotypes after a single dose in most subjects. These results suggest that a more comprehensive study of the immune responses to infection and vaccination in NHP may significantly help identify new immune correlates of protection.

Until molecular correlates of heterotypic and multitypic immunity are comprehensively identified, vaccine-induced protection should be demonstrated in pre-clinical studies, using animals with less genetic variability, studying the quality of the immune response, and using a rigorous and standardize dengue challenge.

## Conflict of Interest Statement

The authors declare that the research was conducted in the absence of any commercial or financial relationships that could be construed as a potential conflict of interest.

## Supplementary Material

The Supplementary Material for this article can be found online at http://www.frontiersin.org/Journal/10.3389/fimmu.2014.00452/abstract
